# Comparison of Clinical, Biochemical and Histological Features between Adult Celiac Patients with High and Low Anti-Transglutaminase IgA Titer at Diagnosis and Follow-Up

**DOI:** 10.3390/nu15092151

**Published:** 2023-04-29

**Authors:** Gloria Galli, Marilia Carabotti, Laura Conti, Silvia Scalamonti, Bruno Annibale, Edith Lahner

**Affiliations:** Department of Medical-Surgical Sciences and Translational Medicine, Sapienza University of Rome, Sant’Andrea Hospital, 00189 Rome, Italy

**Keywords:** celiac disease, anti-tTG IgA, gluten-free diet, antibodies, titer

## Abstract

In adults, celiac disease (CD) diagnosis is based on specific serology (anti-transglutaminase IgA-anti-tTG) and duodenal histology. Evidence is raising the possibility of perform CD diagnosis based only on high anti-tTG titer in children. We aimed to evaluate clinical, histological and biochemical differences between adult patients with high tTG IgA titer (HT) and those with low titer (LT) at CD diagnosis and follow-up. This retrospective study included consecutive adult CD patients divided into two groups: HT (anti-tTG > 10 × ULN) and LT (anti-tTG < 10 × ULN). Clinical, biochemical and histological features were compared between groups at CD diagnosis and at follow-up. A total of 291 patients were included (HT: 47.1%; LT: 52.9%). At CD diagnosis, HT patients showed a non ‘classical’ presentation (*p* = 0.04), Marsh 3C (*p* = 0.005), hypoferritinaemia (*p* = 0.006) and osteopenia/osteoporosis (*p* = 0.04) more frequently than LT patients. A total of 216 patients (HT: 48.6%; LT: 51.4%) performed a follow-up after a median Gluten-free diet of 14 months; HT patients had persistent antibodies positivity (*p* = 0.001) more frequently and GI symptoms (*p* = 0.04) less frequently than LT patients. In conclusion, HT patients presented severe histological damage more frequently at diagnosis, recovering similarly to LT patients after the start of the Gluten-free diet. At follow-up, anti-tTG persisted positive in HT more frequently compared to LT patients, without differences regarding histological recovery and clinical improvement.

## 1. Introduction

Celiac disease (CD) is an immune-mediated enteropathy in which gluten acts as a trigger for small bowel inflammation. CD affects approximately 1% of the Western population and presents with highly variable clinical manifestations, often characterized by nonspecific and subtle symptoms frequently leading to a delayed diagnosis [1]. The only effective treatment is a gluten-free diet (GFD), which must be maintained long-life in order to obtain clinical, biochemical and histological recovery but also to prevent the main disease-specific complications, such as refractory CD, small bowel adenocarcinoma and enteropathy associated T-cell lymphoma (EATL) [2]. In adults, CD diagnosis is based on both specific serology positivity (anti-transglutaminase IgA—anti-tTG; anti-endomysium IgA-EMA) and duodenal histology, with evidence of villous atrophy associated with intraepithelial lymphocytosis and crypt hyperplasia [2]. Differently from adults, pediatric ESPHGAN guidelines support CD diagnosis based only on antibodies assessment with an anti-tTG IgA value of at least 10 times the upper the limit of normal (ULN) associated with anti-endomysium IgA (EMA) positivity [3]. Nowadays, increasing evidence is raising on the possibility to make a CD diagnosis based only on high anti-tTG IgA titer (>10xULN) in the adult CD population as well. The Finnish National Guidelines recommend the no-biopsy approach in their clinical practice and in adult patients [4]; however, this approach has not yet been accepted by the International Guidelines on adult CD patients [5]. A growing number of studies on this matter showed the accuracy of the no-biopsy approach with high specificity and positive predictive value of high anti-tTG IgA titer, while sensibility varies, is slightly lower compared to the duodenal biopsies, and is dependent on the antibodies’ values considered [6,7,8]. Other studies, even if showing a positive association between CD-specific histology and anti-tTG IgA high titer, are more cautious in recommending the no-biopsy approach in all adults with CD diagnosis suspicious [9].

It is important to underline that there are also other causes of villous atrophy and/or crypt hyperplasia not directly associated with CD, such as inflammatory bowel diseases, intestinal parasitosis (i.e., Giardiasis), sartan enteropathy, and common variable immunodeficiency [10]. In all these diseases, the correct final diagnosis is often challenging, requiring physician expertise and multiple associated examinations. In addition, esophagogastroduodenoscopy could be useful to assess other upper GI diseases, including GI tumors, which are slightly increased in CD patients [11]. In these patients, the upper endoscopy, with duodenal but also gastric biopsies, gain an important role in discerning the right diagnosis and excluding other eventually associated differential diagnosis.

Moreover, differently from children, CD adults have a lower rate of histological recovery after the start of a GFD and, consequently, a higher risk of CD complications leading to the necessity of close monitoring of their clinical, biochemical and histological features over time [12,13,14].

In order to understand the possible impact of the no-biopsy approach in adult CD patients, some studies assessed features of patients with high anti-tTG IgA titer at CD diagnosis time, demonstrating that differences between adults with high anti-tTG IgA titer and low tTG titer exist but are scarce [7,15,16,17]. A number of papers were published on these differences in CD children, but only a smaller count exists for adults. It has been demonstrated that patients with high tTG IgA titer at the CD diagnosis time presented more frequently with more severe duodenal histopathology [7], anemia [17] and diarrhea [15,17]. On the other hand, data on the possible differences during follow-up of these groups of patients are very few [18].

The present study aims to evaluate clinical, histological and biochemical differences between adult patients with high anti-tTG IgA titer and those with low titer at CD diagnosis, and follow-ups as well to assess if these disparities persist over time after the start of a gluten-free diet.

## 2. Materials and Methods

This is a retrospective study conducted on consecutive adult patients with a CD diagnosis between 2010 and 2021 and followed up at our tertiary academic center (Sant’Andrea University Hospital, Sapienza, Rome). The criteria used were (i) adult patients with age > 18 years; (ii) definite CD diagnosis based on both duodenal histology and CD-specific antibodies assessment; and (iii) anti-tTG IgA and EMA evaluation before the start of a GFD.

Patients who performed CD-specific diagnostic antibody assessment after the start of a GFD; those with total IgA deficiency; and with incomplete clinical, biochemical, and histological information both at CD diagnosis and at the follow-up were excluded from the study.

All patients who performed the follow-up visits had a GFD compliance evaluation through the Biagi score [19] and clinical/biochemical assessment according to a standardized protocol currently used in our referral center. Histological evaluation was also performed in those patients who performed the control esophagogastroduodenoscopy after the start of a GFD.

The time between the first onset of the GI symptoms/malabsorption signs and the CD diagnosis was assessed and reported at the first visit.

All data from the included CD patients were anonymized to guarantee the secure processing of sensitive data and collected into a predefined spreadsheet. The study was conducted according to the Sapienza Sant’Andrea Hospital protocol, and written informed consent was obtained from all the included patients at the time of CD diagnosis. The study protocol conforms to the ethical guidelines of the 1975 Declaration of Helsinki, as reflected in a priori approval by the institution’s human research committee.

### 2.1. Endoscopic Procedures and Histological Classification

Esophagogastroduodenoscopy (EGDS) with at least four biopsies obtained from the second part of the duodenum was performed in all patients using a flexible video-gastroscope (Olympus GIF-Q165, GIFQ185). As of 2017, to increase the diagnostic yield, bulb biopsies were also collected for the CD diagnosis, according to the guidelines. Biopsies were analyzed after hematoxylin, eosin and CD3 immunohistochemical staining and were assessed using the Marsh classification system modified by Oberhuber [20,21] as follows: the presence of normal villous architecture and >30 intraepithelial lymphocytes\100 enterocytes was defined as Marsh I; the presence of normal villous architecture with >30 intraepithelial lymphocytes\100 enterocytes; and crypt hyperplasia was defined as Marsh II; the presence of both crypt hyperplasia and intraepithelial lymphocytosis associated with villous atrophy was defined as Marsh III, distinguished in A, B or C with mild, moderate or severe atrophy, respectively.

All included patients underwent EGDS at diagnosis while a subgroup performed it for the histological re-evaluation in a period ranging from 12 to 36 months after beginning a GFD. The follow-up EGDS was not proposed for those patients with an inadequate GFD due to the expected lack of histological recovery in this population.

The same expert pathologist in the field of CD examined the duodenal biopsies for each patient both at diagnosis and follow-up. The histological persistence of the above-listed alterations was described and classified as Marsh I, Marsh II or Marsh III (A, B or C).

### 2.2. Serological Assays and DEXA Examination

Anti-transglutaminase (tTG) and anti-endomysium (EMA) IgA antibodies were assessed in all patients both at diagnosis and at the time of the histological re-evaluation. All the included patients had IgA tTG titer measured by ELISA assays in routine clinical use at each laboratory center, including Eu-tTG (Eurospital, Trieste, Italy), Phadi Ab, Thermo Fisher Scientific (Uppsala, Sweden), INOVA Diagnostics (San Diego, CA, USA). Antibodies values were measured according to the laboratory ranges evaluated in each center. An indirect immunofluorescence assay was used to detect IgA EMA in monkey esophageal sections. The commercial indirect immunofluorescence kits used were manufactured according to the laboratory kits used in each center: INOVA Diagnostics, San Diego, CA; Eurospital, Trieste, Italy; and Delta Biological srl, Rome, Italy.

After the start of a GFD, patients were instructed to repeat both anti-tTG and EMA IgA assessment in the same laboratory center in which the serological diagnosis was made or, if not possible, at our university hospital center.

Other blood assays, such as complete-blood cell count, ferritin, total cholesterol, triglycerides, total protein count and albumin were performed using standard laboratory techniques to investigate the presence of associated signs of malabsorption. Values of biochemical alterations were taken into consideration according to their standard laboratory ranges.

### 2.3. Clinical Assessment and GFD Evaluation

Presence, frequency and intensity of GI symptoms were assessed at diagnosis and GFD follow-up through a standardized questionnaire currently used in our department [22,23], including the Bristol scale [24]. Upper GI symptoms, such as vomiting/nausea, heartburn, regurgitation, dysphagia and troublesome postprandial fullness/early satiety, were considered if they were present at least once a week for at least the last three months [25]. Lower GI symptoms, abdominal pain and troublesome abdominal bloating were considered if they were present with at least a weekly frequency; constipation was defined as fewer than 3 spontaneous bowel movements per week or straining, with lumpy hard stools (Bristol scale 1–2); and diarrhea was defined as increased frequency (>3 stools/day) or decreased consistency (loose or liquid stools, Bristol scale 6–7) of bowel movements for at least 3 months before the CD diagnosis [26]. GI symptoms were compared before and after the start of a GFD to evaluate possible clinical changes after the start of a GFD.

According to the Oslo definition [27], the term ‘classical CD’ define patients presenting, at the diagnosis time, signs and symptoms of malabsorption, always including at least diarrhea, steatorrhea or weight loss.

Biagi score [19] consists of four questions about how patients managed their GFD (0–2 = voluntary gluten ingestion, not adequate GFD; 3–4 = adequate GFD); this score was administered by two dedicated physicians during follow-up visits. Patients were also instructed and specifically interviewed by two dedicated physicians to rule out gluten occult contaminations both at the CD diagnosis and at the follow-up.

### 2.4. Statistical Analyses

Descriptive statistics are expressed as numbers, percentages (%) of totals and medians (ranges). Univariate analyses were performed by t-test, Fisher’s exact test and/or chi-squared test for continuous or categorical variables to identify differences between CD patients with High or Low anti-tTG IgA titer. Two-tailed *p* values < 0.05 were considered statistically significant. Statistical analyses were performed by MedCalc© Statistical (MedCalc Software bv, Ostend, Belgium).

## 3. Results

**Figure 1:**
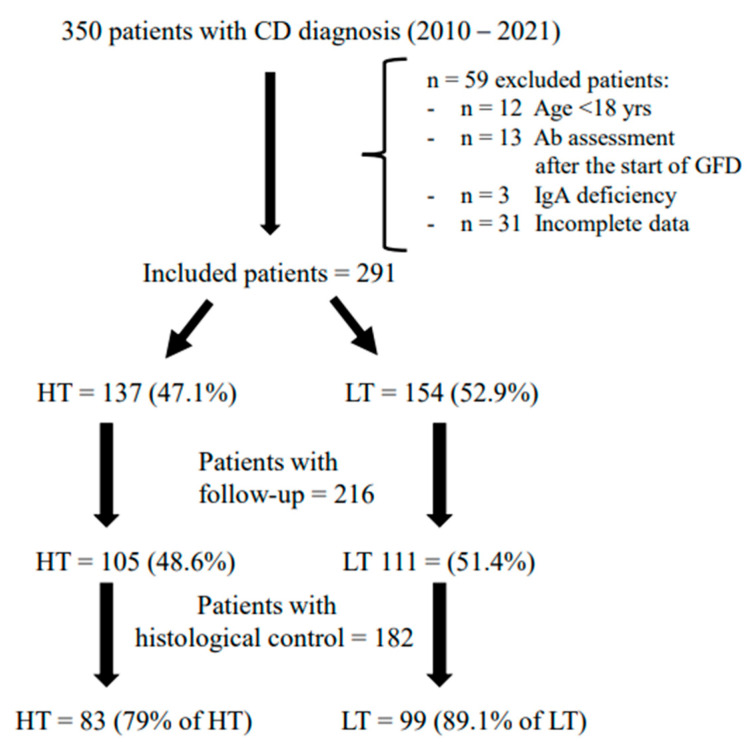
Flow-chart of the studied population. Legend: CD: Celiac Disease; HT: High Titer anti-tTG IgA; LT: Low Titer anti-tTG IgA.

A total of 291 (Females 71.1%; median age 38; range 18–76 years) with CD diagnosis performed between 2010 and 2021 were included in the study. Figure 1 represents the flowchart of the studied population. All the included patients performed anti-tTG IgA assessment before the start of a GFD and were divided into two different groups according to the antibody’s values: high titer group (HT) with tTG IgA value > 10 times upper the limit of normal (ULN) and low titer group (LT) with tTG value < 10 times ULN. According to this definition, 47.1% (females 75.2%; median age 38, range 18–72 years) had HT while 52.9% (Females 67.5%; median age 38, range 18–76 years) presented LT.

In Table 1, the clinical, histological, and biochemical characteristics and a comparison between groups at CD diagnosis time are summarized. HT anti-tTG IgA patients presented a ‘classical’ CD presentation (such as diarrhea, abdominal pain, and weight loss associated with signs of malabsorption) less frequently than LT patients (23.5% vs. 35.1%; *p* = 0.039). Contrarily, HT patients exhibited the more severe histological damage Marsh IIIC (67.1% vs. 47.4%; *p* = 0.005) and osteopenia/osteoporosis (63.5% vs. 50%; *p* = 0.04) more frequently than patients with LT. Concerning GI symptoms, considering both upper and lower GI symptoms, only regurgitation was significantly more prevalent in patients with LT (*p* = 0.01). Regarding signs of malabsorption, compared to LT patients, HT patients showed hypoferritinaemia (66.6% vs. 45.2%; *p* = 0.006) significantly more frequently with a higher percentage of anemia (both microcytic, normocytic and macrocytic) without reaching statistical significance (45.9% vs. 35.3%; *p* = 0.07). Both the presence of hypocholesterolemia and hypotriglyceridemia showed comparable results between the two groups. No other significant differences were found between the HT and LT groups concerning gender, age, BMI, autoimmune comorbidities, duration of symptoms/signs before CD diagnosis and the other GI symptoms, and signs of malabsorption.

After a median of 14 months (range 12–36 months) of a GFD, 216 patients (74.2% of the total) performed the clinical follow-up. The HT anti-tTG IgA group consisted of 48.6% of patients while the LT group of 51.4%. Table 2 describes the characteristics of groups after the GFD follow-up. GFD, assessed through the Biagi score, was described as adequate (score 3–4) in a widely high percentage of patients, without statistical differences between the two groups (95% and 91.8% in HT and LT, respectively, *p* = 0.41). Additionally, the duration of the GFD follow-up was similar between groups (with a median of 14 months in both groups; *p* = 0.43). A total of 84.1% of patients (HT 79% and LT 89.1%) also performed the endoscopic/histological control after at least 12 months of a GFD and no statistically significant differences were detected between groups concerning adherence to the follow-up EGDS (*p* = 0.06). At the GFD follow-up, HT patients had persistent antibodies positivity (34.3% vs. 14.4%; *p* = 0.001) more frequently compared to LT patients. In particular, considering only patients with persistent antibodies positivity after a GFD, 29.4% of HT and 33.3% of LT patients only had tTG IgA positivity with EMA IgA negativity, while 20.6% of HT and 20% of LT presented with EMA positivity and with anti-tTG negativity. The other 50% and 46.7% of HT and LT patients, respectively, presented both tTG and EMA IgA positivity.

Concerning GI symptoms, they were globally significantly less frequent in HT patients (24.3% vs. 38.2%; *p* = 0.04), in particular abdominal pain which was statistically less represented in this group compared to the LT group (*p* = 0.047). Regarding histological recovery (both persistent duodenal atrophy and mucosal healing) and signs of malabsorption, there were no significant differences between the two groups.

## 4. Discussion

CD-specific serology assessment has always influenced the scientific debate on CD diagnosis. The advent of anti-tTG IgA antibodies has changed the diagnostic approach in CD patients, allowing an easier and more accurate diagnosis [28,29]. Recently, several studies reported a quite high sensibility and specificity of anti-tTG IgA in detecting CD without the necessity of histological evaluation in adult patients [9,30]; however, in the literature, there are some results about the potential clinical, histological and biochemical differences between patients with high titer and those with low titer at the time of CD diagnosis; only one study evaluated these differences after the start of a GFD [18].

In this study, we showed that, at the time of CD diagnosis, adult patients with HT anti-tTG IgA presented a more severe disease with a higher rate of total villous atrophy (Marsh 3C), hypoferritinaemia and osteopenia/osteoporosis than patients with LT. Other studies showed the correlation between anti-tTG IgA titer and histological damage arguing that the autoimmune response is strictly involved in the intestinal inflammation and could be directly associated with the severity of the duodenal damage [7,16,17,30,31,32].

Hypoferritinaemia is an important and frequent sign of malabsorption in newly diagnosed celiac patients [33]. In line with a more severe disease, we documented an increased frequency of this abnormality in HT patients than in those with LT. This could also be due to the higher rate of total villous atrophy detected in these patients. One study observed the worsening of mean hemoglobin values with the increase in the anti-tTG IgA titer [17] while another one documented an increased presence of microcytosis in HT anti-tTG IgA patients without differences in median ferritin and hemoglobin values [18].

Concerning the presence of osteopenia/osteoporosis, this is thought to be another factor influenced by malabsorption and chronic inflammation in CD [34]. Consequently, both the presence of total villous atrophy and the increase in the specific antibodies could explain the increased prevalence of osteopenia/osteoporosis in HT anti-tTG patients. We can speculate that in HT anti-tTG CD patients, the BMD (Bone Mineral Density) assessment through a DEXA scan could be useful in order to identify the presence of BMD alterations early on. This feature has been poorly assessed in other studies [15], with only one study finding no association with anti-tTG IgA values [17]. It follows that other studies are needed to evaluate this topic.

Even if CD classical presentation has been demonstrated more frequently in our LT anti-tTG IgA patients compared to the HT group, no statistically significant differences were found concerning the global presence of GI symptoms. In fact, even if LT patients presented a slightly higher percentage of global GI symptoms, only regurgitation was significantly more prevalent in patients with LT. These results are in line with the controversial data described in other studies, in which no association has been found between GI symptoms frequency and severity of the duodenal damage at the time of CD diagnosis [35,36,37]. Only a few studies reported this association [15]. Specifically, regarding the association between anti-tTG IgA titer and GI symptoms at the time of CD diagnosis, only one study documented a quite high rate of symptoms in patients with LT anti-tTG IgA [18]. To explain this result, we can speculate that patients with important and suggestive GI symptoms receive a CD diagnosis more easily and earlier compared to those with a low rate of symptoms, in which the perpetuation of inflammation could consequently cause an increase in CD-specific autoantibodies.

After a similar median time of the GFD follow-ups, the two groups of patients showed very few differences. Persistent positive CD-specific antibodies (tTG IgA and/or EMA) have been demonstrated to be more frequent in patients with higher anti-tTG IgA titer at the time of CD diagnosis (Table 2). Differently, histological recovery and persistence of duodenal atrophic disease were similar between groups with a comparable rate of adequate GFD compliance, demonstrating that antibody positivity does not strictly reflect histological recovery. It has already been demonstrated that antibody positivity persistence is not always associated with persistent symptoms and/or histological damage [38,39]. Therefore, we can speculate that the persistence of CD-specific antibody positivity, in patients with HT, could be also associated with the detected antibodies’ initial values with a slow serological reduction, thereby not allowing us to only rely on specific antibodies for the histological healing assessment after the start of a GFD.

Regarding the presence of GI symptoms at the time of the GFD follow-up, even if an important reduction has been observed in both groups, it was found to be globally more frequent in patients with LT anti-tTG IgA than HT, and in particular, with the presence of abdominal pain. One study showed the increased prevalence of GI symptoms at the time of CD diagnosis in patients with LT anti-tTG IgA but, differently from our results, lacks any findings of symptomatic differences between HT and LT groups at the time of a GFD follow-up [18]. We previously demonstrated the high percentage of GI symptoms in CD patients on a GFD, even in the absence of persistent duodenal mucosal damage [23]. On one hand, we can assert that the presence of both persistent CD-specific antibodies and duodenal mucosal damage are not associated with GI symptoms at the time of the GFD follow-up; on the other, we confirm that, in CD patients on GFD, GI symptoms are not always associated with CD while also considering other possible diagnoses such as irritable bowel disease, reflux syndrome, functional dyspepsia, etc.

Signs of malabsorption were similarly detected in both groups of patients at the GFD follow-up. This means that differences identified at the time of CD diagnosis were solved overtime according to the duodenal mucosal damage and independently from the anti-tTG IgA initial values. Approximately one-third of patients of both groups continued to present hypoferritinaemia, which may be due to a slow recovery of the iron deposits and/or unrelated to CD, as gynecologic disorders (polymenorrhea) in female patients have yet to be described in other studies [40].

As already discussed, histological recovery was similar between groups, demonstrating that even if patients with HT anti-tTG IgA presented with more severe disease at the time of CD diagnosis, the duodenal damage was resolved, similarly to patients with less frequent severe damage.

The major limitation of the study is the retrospective analysis of the data. This possible limitation could be overcome by the strict methodology used both for the assessment of features at the time of CD diagnosis and the analysis of these data, which has been stringent for all the patients, in line with the exclusion criteria declared. Another limitation is the lack of use of alternative methods for GFD assessment such as urine- or fecal-gluten peptide examination for all included patients. These methods were not evaluated systematically in all patients due to their availability after the beginning of the study patient inclusion. We have tried to overcome this limit by improving the GFD adherence evaluation through a careful interview performed by two dedicated physicians

A strength of this study is the standardized clinical, biochemical and histological evaluation with a high number of patients assessed, including during the GFD follow-ups and with the histological re-evaluation. In fact, to our best knowledge, very few studies have assessed HT anti-tTG IgA patients after the start of a GFD.

In conclusion, our results demonstrated that, at the time of CD diagnosis, patients with HT anti-tTG IgA presented a slightly more severe disease than patients with LT anti-tTG IgA in terms of histology and signs of malabsorption but not for GI symptoms. These features recovered similarly in the two groups after the start of a GFD, suggesting that HT tTG IgA presence at the time of CD diagnosis could not influence the clinical, biochemical and histological follow-up. Other studies with prospective design and longer GFD follow-ups are needed to confirm this result.

It is however important to underline that, at the time of GFD follow-up, neither specific antibodies nor clinical symptoms were predictive of mucosal recovery, which remains a very important goal for the CD patients’ management. In fact, the lack of duodenal histological recovery is the basic condition for CD complications development, such as refractory CD or neoplastic complications. Therefore, we should differentiate patients who gained a partial histological recovery after the start of the GFD (labelling the patients as having ‘slow recovery’) to those patients with persistent atrophic damage with any recovery of the duodenal mucosa in order to understand the potential curative effect of the diet [11,35]. For this purpose, the knowledge of diagnostic duodenal mucosal histology could remain an important supporting tool for CD patients’ follow-up management.

## Figures and Tables

**Table 1:** Features and comparison between High anti-tTG IgA titer (HT) and Low anti-tTG IgA titer (LT) patients at celiac disease diagnosis time.

	HT Patients with Ab > 10 ULN *n* = 137 (47.1%)	LT Patients with Ab < 10 ULN *n* = 154 (52.9%)	*p* Value
Females	75.2%	67.5%	0.156
Median age, years (range)	38 (18–72)	38 (18–76)	0.857
Median BMI kg/m^2^ (range)	21.8 (15.9–33.8)	22.5 (16.4–38.2)	0.218
Underweight (BMI < 18 kg/m^2^)	12.8%	6.9%	0.728
Autoimmune comorbidities	30.6%	31.8%	0.899
Family history of CD	17.5%	18.5%	0.878
>3 years duration of symptoms/signs before the CD diagnosis	42.2%	34.1%	0.194
Marsh IIIC	67.1%	47.4%	0.005
Clinical presentation Classical presentation	23.5%	35.1%	0.039
GI symptoms Total pts with GI symptoms Nausea/Vomit Heartburn Regurgitation Dysphagia Postprandial fullness/early satiety Abdominal pain Abdominal bloating Constipation Diarrhoea	73.1% 19.4% 26.1% 13.4% 8.2% 37.3% 43.6% 51.5% 14.2% 22.4%	82.5% 24.7% 31.2% 25.3% 11% 43% 50% 58.4% 15.6% 27.9%	0.063 0.321 0.363 0.012 0.434 0.151 0.288 0.285 0.743 0.341
**Signs of malabsorption** Total pts with signs of malabsorption Anaemia Hypoferritinaemia Hypocholesterolaemia Hypotriglyceridaemia	68.6% 45.9% 66.6% 7.7% 6.1%	67.7% 35.3% 45.2% 13% 8%	0.899 0.071 *0.006* 0.220 0.629
**Osteopenia/Osteoporosis**	63.5%	50%	*0.039*

Legend: ULN: upper the limit of normal; BMI: body mass index; CD: celiac disease; GI: gastrointestinal.

**Table 2:** Features and comparison between High anti-tTG IgA titer (HT) and Low anti-tTG IgA titer (LT) patients at the time of GFD follow-up.

	HT Patients with Ab > 10 ULN n = 105 (48.6%)	LT Patients with Ab < 10 ULN n = 111 (51.4%)	*p* Value
**Females**	76.2%	65.8%	0.101
**Median months of GFD (range)**	14 (12–36)	14 (12–36)	0.435
**Adequate GFD**	95%	91.8%	0.414
**Median BMI kg/m^2^ (range)**	22.4 (16.3–32.8)	23 (20.5–36.6)	0.284
**Underweight (BMI < 18 kg/m^2^)**	5.3%	3.9%	0.739
**GI symptoms** Total pts with GI symptoms Nausea/Vomit Heartburn Regurgitation Dysphagia Postprandial fullness/early satiety Abdominal pain Abdominal bloating Constipation Diarrhoea	24.3% 1% 5.8% 2.9% 0 3.9% 9.6% 11.6% 5.8% 4.8%	38.2% 3.7% 9.2% 5.6% 1.8% 7.3% 18.3% 21.1% 10.1% 8.2%	*0.038* 0.370 0.439 0.499 0.376 *0.047* 0.067 0.315 0.410
**Antibodies positivity**	34.3%	14.4%	*0.001*
**Signs of malabsorption** Total pts with signs of malabsorption Anaemia Hypoferritinaemia Hypocholesterolaemia Hypotriglyceridaemia	29.1% 13.7% 28.4% 2% 11.6%	33.9% 11.2% 28.1% 3.9% 8%	0.464 1 1 0.684 0.472
**Hystological control** **Marsh III (atrophic disease)** **Marsh 0 score**	79% 33.3% 54.7%	89.1% 24.2% 58.6%	0.060 0.191 0.654

Legend: **ULN**: upper the normal limit; **GFD**: Gluten-free diet; **BMI**: Body Mass Index; **GI**: Gastrointestinal.

## Data Availability

The data presented in this study are available upon request from the corresponding author. The data are not publicly available due to ethical restrictions.
